# Diabetes mellitus affects the biomechanical function of the callus and the expression of TGF-beta1 and BMP2 in an early stage of fracture healing

**DOI:** 10.1590/1414-431X20154736

**Published:** 2015-11-27

**Authors:** M.T. Xu, S. Sun, L. Zhang, F. Xu, S.L. Du, X.D. Zhang, D.W. Wang

**Affiliations:** 1 Department of Joint Surgery, Shandong Provincial Hospital, Shandong University, Jinan, Shandong Province, China; 2 Department of Orthopaedics, Liaocheng People's Hospital, Liaocheng Clinical School, Taishan Medical University, Liaocheng, Shandong Province, China; 3 Central Laboratory, Liaocheng People's Hospital, Liaocheng Clinical School, Taishan Medical College, Liaocheng, Shandong Province, China; 4 Department of Pathology, Liaocheng People's Hospital, Liaocheng Clinical School, Taishan Medical University, Liaocheng, Shandong Province, China

**Keywords:** Diabetes mellitus, biomechanical function, TGF-beta1, BMP-2, fracture healing, Mechanical parameters

## Abstract

Transforming growth factor beta 1 (TGF-β1) and bone morphogenetic protein-2 (BMP-2) are important regulators of bone repair and regeneration. In this study, we examined whether TGF-β1 and BMP-2 expressions were delayed during bone healing in type 1 diabetes mellitus. Tibial fractures were created in 95 diabetic and 95 control adult male Wistar rats of 10 weeks of age. At 1, 2, 3, 4, and 5 weeks after fracture induction, five rats were sacrificed from each group. The expressions of TGF-β1 and BMP2 in the fractured tibias were measured by immunohistochemistry and quantitative reverse-transcription polymerase chain reaction, weekly for the first 5 weeks post-fracture. Mechanical parameters (bending rigidity, torsional rigidity, destruction torque) of the healing bones were also assessed at 3, 4, and 5 weeks post-fracture, after the rats were sacrificed. The bending rigidity, torsional rigidity and destruction torque of the two groups increased continuously during the healing process. The diabetes group had lower mean values for bending rigidity, torsional rigidity and destruction torque compared with the control group (P<0.05). TGF-β1 and BMP-2 expression were significantly lower (P<0.05) in the control group than in the diabetes group at postoperative weeks 1, 2, and 3. Peak levels of TGF-β1 and BMP-2 expression were delayed by 1 week in the diabetes group compared with the control group. Our results demonstrate that there was a delayed recovery in the biomechanical function of the fractured bones in diabetic rats. This delay may be associated with a delayed expression of the growth factors TGF-β1 and BMP-2.

## Introduction

There is strong evidence indicating that patients with diabetes mellitus type 1 (DM1) have decreased bone mass and are at increased risk of fragility fractures (1 2 3 4 5). It is also established that defects in osteoblast differentiation and activity are the main factors underlying bone fragility in DM1 (6 7 8). Other contributing factors include an accumulation of advanced glycation end products (AGEs) and the development of diabetes complications, such as neuropathy and hypoglycemia, which cause a further decline in bone mineral density (5). Several experimental and clinical studies have documented an association between diabetes and impaired bone healing (1,5,9,10). The existence of an elevated fracture risk in diabetes suggests the involvement of pathogenic influences, such as hyperglycemia, on bones (1). Macey et al. (3) showed that the fracture callus from untreated diabetic rats exhibited 29% lower tensile strength and 50% decreased stiffness compared with non-diabetic rats 2 weeks after the production of a closed fracture. Herbsman et al. (4) found a significant reduction in the tensile strength of a fibular fracture in an alloxan-induced diabetic rat model at 4 weeks post-fracture. Diabetes-associated conditions affect bone mass and bone quality (11,12). Experimental studies have suggested that the malfunction of bone marrow-derived osteoclasts may contribute to decreased cartilage resorption and delayed endochondral ossification in diabetes (13). However, the mechanisms of delayed fracture healing in diabetic patients or animal models remain unclear. Transforming growth factor beta 1 (TGF-β1) and bone morphogenetic protein-2 (BMP-2) are two very important factors in the process of bone healing (14 15 16 17 18 19). However, little is known about the expression of TGF-β1 and BMP2 in the bone healing processes of patients with diabetes.

## Material and Methods

### Diabetes model

The animal protocol for this investigation was approved by the Animal Ethics Committee of Liaocheng People's Hospital, China. A total of 190 adult male Wistar rats of 10 weeks of age (purchased from the Animal Experimental Centre of Medical Department of Shandong University, China) were used in this study. Rats were divided into two groups (n = 95 each), a control group and a diabetes group. The animals were housed in groups, fed a diet of standard laboratory feed and were weighed daily for 7 days. Diabetes was induced in test rats by intraperitoneal injection of 160 mg/kg alloxan and was defined as blood glucose levels of more than 16.7 mmol/L, which was determined 2 days after alloxan injection. If the desired glucose level was not achieved, an additional injection was given to achieve the appropriate level. Control group rats (n=95) received intraperitoneal injection of placebo and served as controls.

### Fracture model

All studies were performed in rats with diabetes for 3 weeks prior to fracture. Before the surgery, all animals, control and diabetic, were weighed and anesthetized with 100 mg/kg ketamine. A 10×2.5×1.5 mm fracture was created in the middle of the left tibia in all rats using a rongeur (Wego Ortho, China). All rats were injected intramuscularly with penicillin (10,000 units) 3 days post-operation for infection prevention. Fractures were allowed to heal for 5 weeks.

### Biomechanical function measurement

Forty-five rats from each group were sacrificed by cervical dislocation at 3, 4, and 5 weeks post-fracture. The mechanical testing parameters were obtained by the measurement of five animals in each group. The tibias were resected, stripped of the surrounding soft tissues, and evaluated for bone healing by manual palpation. The proximal and distal ends of the tibia were embedded in wood's metal alloy blocks, and the diaphysis was wrapped in gauze soaked with normal saline. The tibias were then mounted on a combined axial motion and torsional testing jig that was attached to a biaxial material testing machine (MTS 858 Bionix, MTS Systems, USA). Three mechanical testing parameters were measured in both diabetic and control rats. The tibias were mounted so that vertically directed loads would create a three-point bending. The samples were tested to failure in displacement control at a rate of 15 mm/min using a biaxial material testing machine (MTS 858 Bionix). Failure was defined as the maximum force of callus strength recorded during testing. A torque test was conducted to failure at a rate of 15 mm/min. The bent rigidity, torsional rigidity, and destruction torque were automatically calculated using MTS 858 Bionix software.

### Histomorphometry studies

At 1, 2, 3, 4, and 5 weeks post-fracture, five rats were sacrificed from each group. Twenty-five tibia samples were extracted and processed for histology by fixation in 10% neutral-buffered formalin for 24 h at 4°C. After washing with phosphate-buffered saline (PBS) overnight, the tissues were decalcified with 0.36 mol/L ethylene diamine tetraacetic acid (EDTA; pH 7.0-7.2) and embedded in paraffin. The samples were cut into ∼5 µm-thick sagittal sections using a low-speed diamond saw. The sections were then glued to microscope slides. The slides were stained with hematoxylin and eosin, Alcian blue, or immunohistochemical stains. Immunohistochemistry was performed using the avidin-biotin peroxidase complex method. An SABC kit (purchased from Wuhan Boster Biological Technology Ltd., China) was used for immunostaining with polyclonal antibodies against BMP-2 (antibody BA0290) and TGF-β1 (antibody BA0290). After deparaffinization and hydration, the specimens were treated with 0.01% trypsin (Sigma, USA) for 30 min at 37°C. Endogenous peroxidase was blocked with methanol containing 0.3% hydrogen peroxide. Sections were incubated with PBS containing 5% normal goat serum and 1% bovine serum albumin for 30 min at room temperature to eliminate nonspecific binding, followed by the appropriate concentrations of primary antibodies at 4°C overnight in a humidified chamber. After washing with PBS three-times for 5 min, sections were incubated with a biotinylated secondary antibody and an avidin-biotin peroxidase complex in a humidified chamber for 30 min at room temperature. Color was developed using 3, 39-diaminobenzidine tetrachloride (Dojindo Chemical Laboratories, Japan). Finally, the sections were counterstained with hematoxylin. As negative controls, PBS, normal rabbit IgG, or normal mouse IgG were used instead of the primary antibodies. Gray scale images of TGF-β1 and BMP2 were automatically recorded using Leica-QwinV3 image analysis software (Germany).

### Quantitative reverse-transcription polymerase chain reaction (qRT-PCR)

Twenty-five tibial samples were homogenized in a liquid nitrogen-cooled mortar. Tissue powders were then processed for RNA extraction using the TriZol method. Using the SuperScript™ first-strand synthesis system (Invitrogen, USA), 2.5 µg total RNA was reverse-transcribed and the products were treated with RNase H before storing at -20°C. Real-time PCR was performed on a real-time PCR detection system (MJ, Research Opticon, USA). Using SYBR¯ green PCR Master Mix reagents (Bio-Rad Laboratories, USA), each reaction mixture consisted of 12.5 µL SYBR green PCR reagent, 2.5 µL of 1:50 diluted reverse-transcription product, 5 mM of primers, and diethylpyrocarbonate-treated water, in a total volume of 25 µL. No template and non-reverse-transcribed reactions were included in each PCR plate as negative controls. 18S was used as an internal standard in each PCR plate. After 10 min at 95°C, the PCR amplification was performed for 40 cycles. Each cycle consisted of amplification at 95°C for 50 s and 65°C for 30 s. The 7900 HT Real-Time PCR System (Applied Biosystems, USA) was used to perform the reaction. The expression level was normalized against endogenous GAPDH for related gene expression. Primer sequences for qPCR are listed in Table 1.

**Table:**
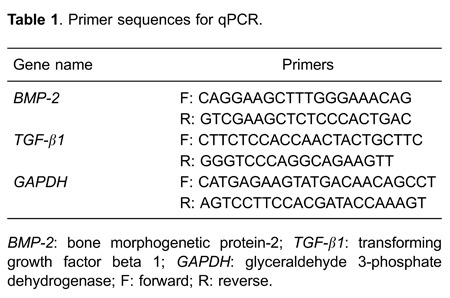

### Statistical analysis

The experimental data were analyzed using SPSS10. The paired designed T-test was used to compare differences between groups. All data are reported as means±SE, and differences were considered statistically significant when P<0. 05.

### Results

#### Biomechanical function

**Figure 1:**
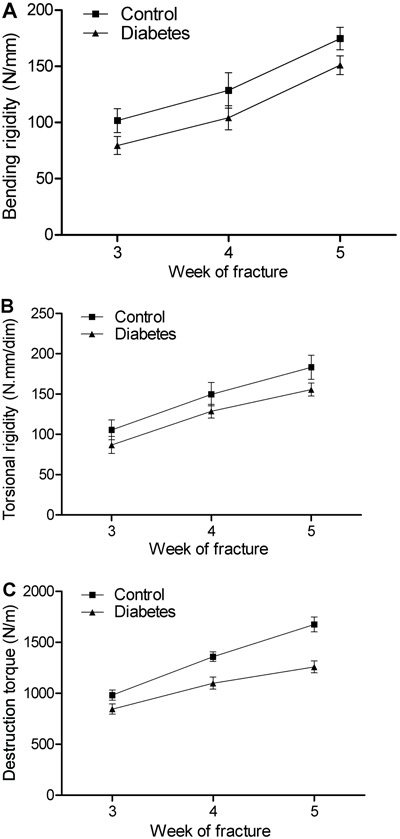
At each time point, the mean bending rigidity (*A*), mean torsional rigidity (*B*), and mean destruction torque (*C*) in the control group was greater compared to the diabetes group (P<0.05, *t*-test). The means of five independent experiments are shown for all conditions.

The mechanical parameters (bending rigidity, torsional rigidity, destruction torque) of the control and diabetes groups increased continuously throughout the experimental period; however, two-way ANOVA revealed that the diabetes group had significantly lower mean values for the bending rigidity, torsional rigidity and destruction torque than the control group P<0.05 (Figure 1). Using biaxial material machine testing, bone biomechanical functional recovery was well characterized by the three mechanical testing parameters.

#### TGF-β1 histochemistry and immuno-histochemistry finding

**Figure 2:**
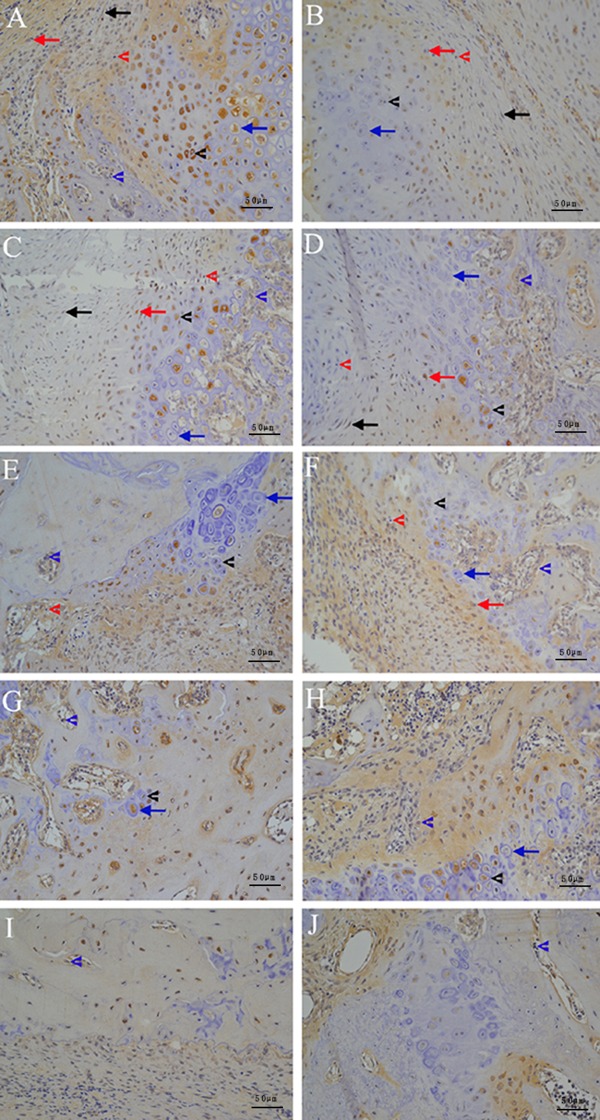
Local expression of transforming growth factor β1 (TGF-β1) during fracture healing is shown in the control (*A*) and diabetes (*B*) groups after 1 week of healing, 2 weeks of healing (*C*) and (*D*), 3 weeks of healing (*E*) and (*F*), 4 weeks of healing (*G*) and (*H*), and 5 weeks of healing (*I*) and (*J*), respectively (DAB ×200). Red arrows: osteogenic cells; red arrowheads: osteoblasts in the subperiosteal bone; black arrows: fibroblast-like cells; black arrowheads: proliferating chondrocytes; blue arrows: mature chondrocytes; blue arrowheads: osteoblasts in the trabecular bone near to endochondral ossification front.

The local expression of TGF-β1 during an early stage of healing is shown in Figure 2. In the control group at week 1 post-fracture, a moderate staining for TGF-β1 was seen in osteogenic cells, fibroblast-like cells, chondrocytes, and osteoblasts in both the subperiosteal bone and the trabecular bone near the endochondral ossification front. In the diabetes group, moderate staining for TGF-β1 was only seen in osteogenic cells, with weak staining in osteoblasts in both the subperiosteal bone and the trabecular bone near the endochondral ossification front. At week 5, in control group, moderate staining for TGF-β1 was seen in osteoblasts in both the subperiosteal bone and the trabecular bone near to the endochondral ossification front, with weak staining in osteogenic cells. In the diabetes group, moderate staining for TGF-β1 was seen in osteoblasts in the subperiosteal bone and weak staining was seen in osteogenic cells. No staining was seen in hypertrophic chondrocytes in control or diabetic rats.

**Figure 3:**
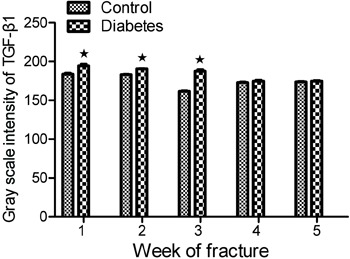
Gray scale intensity of transforming growth factor β1 (TGF-β1). The gray scale intensity value of TGF-β1 expression was higher in the diabetes group than in the control group at postoperative weeks 1, 2, and 3. The difference between groups was significant (*P<0.05, *t*-test).

The gray scale intensity of TGF-β1 expression was higher in the diabetes group than in the control group, with the differences being statistically significant (P<0.05) at postoperative weeks 1, 2 and 3. However, there was no statistically significant difference (P>0.05) at weeks 4 and 5. The gray scale intensities of TGF-β1 expression are shown in Figure 3.

#### BMP-2 histochemistry and immuno-histochemistry findings

**Figure 4:**
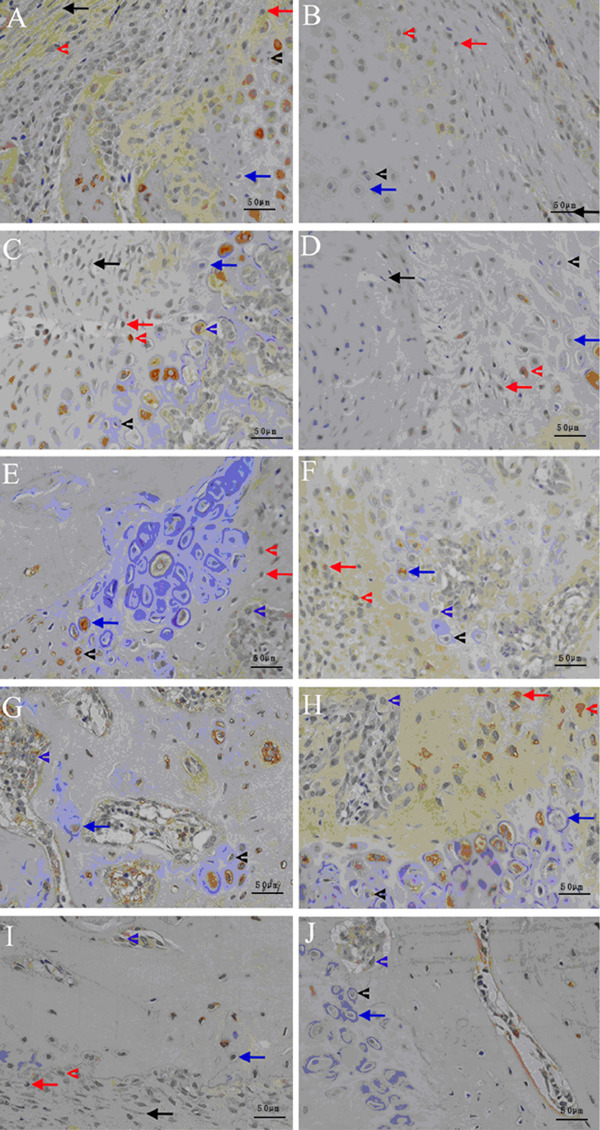
Local expression of bone morphogenetic protein-2 (BMP-2) during fracture healing is shown in the control (*A*) and diabetes (*B*) groups after 1 week of healing, 2 weeks of healing (*C*) and (*D*), 3 weeks of healing (*E*) and (*F*), 4 weeks of healing (*G*) and (*H*), and 5 weeks of healing (*I*) and (*J*), respectively (DAB ×200). Red arrows: osteogenic cells; red arrowheads: osteoblasts in the subperiosteal bone; black arrows: fibroblast-like cells; black arrowheads: proliferating chondrocytes; blue arrows: mature chondrocytes; blue arrowheads: osteoblasts in the trabecular bone near to endochondral ossification front.

The local expressions of BMP-2 during an early stage of healing are shown in Figure 4. At week 1 post-fracture, in the control group, moderate staining for BMP-2 was seen in osteogenic cells, fibroblast-like cells or chondrocytes, osteoblasts in the subperiosteal bone, proliferating chondrocytes and osteoblasts in the trabecular bone near the endochondral ossification front. In the diabetes group, weak staining for BMP-2 was seen in osteogenic cells, osteoblasts in the subperiosteal bone, proliferating chondrocytes and osteoblasts. At week 5 post-fracture, in the control group, moderate staining for BMP-2 was seen in osteoblasts in the trabecular bone near the endochondral ossification front and weak staining was seen in osteoblasts in the subperiosteal bone and proliferating chondrocytes. In the diabetes group, weak staining for BMP-2 was only seen in osteoblasts in the subperiosteal bone and proliferating chondrocytes. No staining was seen at hypertrophic chondrocytes in controls and diabetic rats.

**Figure 5:**
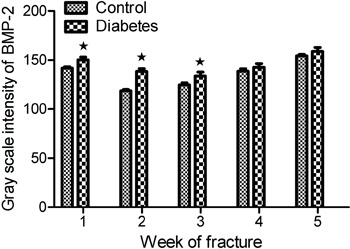
Gray scale intensity of bone morphogenetic protein-2 (BMP-2) in the two groups. The gray scale intensity value of BMP-2 expression was higher in the diabetes group than in the control group at postoperative weeks 1, 2, and 3. The differences between the two groups was significant (*P<0.05, t-test).

The gray scale intensity of BMP-2 expression was higher in the diabetes group than in the control group, with the differences between the two groups being statistically significant (P<0.05) at postoperative weeks 1, 2, and 3. However, there was no statistically significant difference (P>0.05) at weeks 4 and 5. The gray scale intensities of BMP-2 expression are shown in Figure 5.

#### RT-PCR

**Figure 6:**
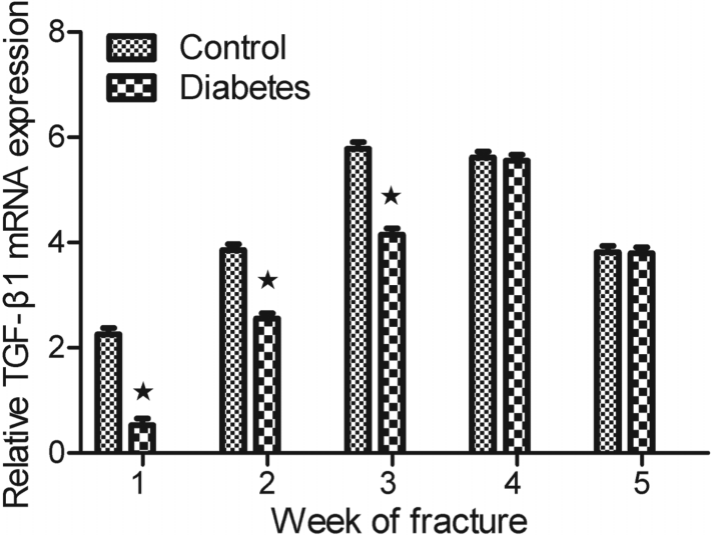
Fluorogenic quantitative polymerase chain reaction results of transforming growth factor β1 (TGF-β1) mRNA expression in the two groups. TGF-β1 gene expression in the diabetes group was lower than in the control group at postoperative weeks 1, 2, 3 (*P<0.05, *t*-test). However, there was no statistical difference at weeks 4 and 5. TGF-β1 expression achieved its highest level at week 4 in the diabetes group, but reached its highest level at week 3 in the control group.

The results of RT-PCR for TGF-β1 expression (Figure 6) suggest that TGF-β1 mRNA expression in the diabetes group was lower than in the control group at postoperative weeks 1, 2, 3(P<0.05). However, there was no statistical difference at week 4 and 5. TGF-β1 expression achieved its highest level at week 4 in the diabetes group, but reached its highest level at week 3 in the control group. Thus, peak TGF-β1 expression was delayed by 1 week in the diabetes group compared with the control group.

**Figure 7:**
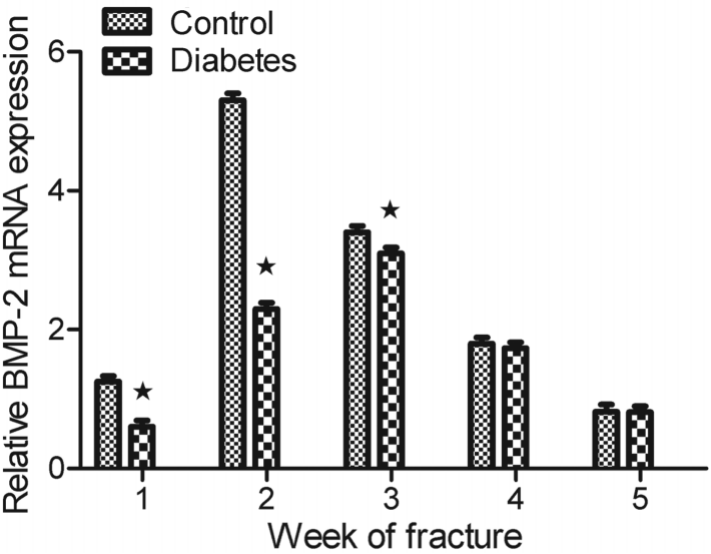
Fluorogenic quantitative polymerase chain reaction results of bone morphogenetic protein-2 (BMP-2) mRNA expression in the two groups. BMP-2 mRNA expression in the diabetes group was lower than in the control group at postoperative weeks 1, 2, 3 (*P<0.05, t-test). BMP-2 expression achieved its highest level at week 3 in the diabetes group, but reached its highest level at week 2 in the control group.

The results of RT-PCR for BMP-2 expression (Figure 7) suggest that BMP-2 mRNA expression in the diabetes group was lower than in the control group at postoperative weeks 1, 2, and 3 (P<0.05). The difference in expression was highest at postoperative week 2. BMP-2 expression achieved its highest level at week 3 in the diabetes group, but reached its highest level at week 2 in the control group. Thus, peak BMP-2 expression was also delayed by 1 week in the diabetes group compared with the control group.

### Discussion

These results indicate distinctive differences in biomechanical functional recovery in the early stages of fracture healing in the diabetes group compared with the control group. Furthermore, the delayed bone functional recovery was well characterized by the mechanical testing parameters in the diabetes group. In the early intramembranous ossification and endochondral ossification stages of fracture-healing, the local expression of TGF-β1 and BMP-2 was strongly increased at an early stage in the control group compared with the diabetes group, in all cells (osteogenic cells, osteoblasts in the subperiosteal bone, fibroblast-like cells, proliferating chondrocytes, mature chondrocytes, and osteoblasts in the trabecular bone near to the endochondral ossification front). The delayed local expression of the growth factors TGF-β1 or BMP-2 coincided with postponed biomechanical functional recovery. These results indicate that diabetes affected fracture healing through the local expression of growth factors in the early stages of healing.

Both clinical and experimental studies have shown that diabetes affects fracture healing (13, 20 21 22). Diabetes affects the biomechanical properties of bone and results in decreased bone mechanical strength (23). In our mechanical experiment, bone functional healing was well characterized by the testing parameters. It showed that diabetes affected the biomechanical properties of bone, resulting in decreased bending stiffness, torsional stiffness and the destruction of torque in the diabetes group. The mechanism by which diabetes affects fracture healing remains unclear. However, the role of cytokines in fracture healing has generated more attention in the scientific community. In the fracture healing process, the expression levels of insulin-like growth factor-1 (IGF-1), insulin-like growth factor-2 (IGF-2), platelet-derived growth factor (PDGF), fibroblast growth factor (FGF) and TGF are decreased in diabetic rats (24,25). TGF-β1 and bone morphogenetic proteins (BMPs) are a group of multifunctional cytokines in the TGF superfamily that can induce mesenchymal cell migration, proliferation, differentiation, eventually leading to cartilage and bone formation (26). TGF-β1 has been shown to have a stimulating effect on bone healing (17-19 27). TGF-β1 regulates the growth and differentiation of bone and cartilage cells, and promotes the expression and influence of many growth factors in bone and cartilage tissue (28). *In vitro* TGF-β1 and BMP-2 exhibit a strong ability to promote osteoblast differentiation and the induction of ossification (29). TGF-β and BMP-2 could directly promote the induction of bone resorption by mature osteoblasts (30 31 32). In our experiment, we found the local expression of TGF-β1 and BMP-2 appeared in the intramembranous ossification and in endochondral ossification; however, the local expression was lower in the diabetes group than the control group. This suggested that diabetes might inhibit the synthesis of TGF-β1 and BMP-2 and, consequently, affect the formation of bone and cartilage. Furthermore, the change in the local expression of TGF-β1 and BMP-2 may cause the synthesis and local expression of other cytokines, resulting in an inhibition of new bone formation and bone reconstruction, eventually leading to slower fracture healing.

In diabetes mellitus, hyperglycemia or insulin can influence the production of pro-inflammatory or anti-inflammatory cytokines, which can affect proliferation, migration and differentiation of callus cells, in particular osteoblasts, or other blood cells (33). Consequently, delayed local expression of TGF-β1 and BMP-2 further affects the maturation of osteoblasts and cartilage cells. This reduces intramembranous ossification, endochondral bone formation, and matrix synthesis. Our results support the treatment of bone fractures in diabetic rats using cell growth factors (34). However, this should be done at an early stage of fracture healing.

The mechanism by which the local expression of BMP-2 and TGF-β1 is delayed in experimental diabetes is complex. Unlike the acute inflammatory phase of normal healing, diabetics exhibit chronic moderate inflammation (35) that reduces the number and impairs the function of activated macrophages and leads to reduced growth factor levels (36). Several cellular mechanisms have been proposed, including depletion or dysfunction of polymorphonuclear leukocytes and macrophages, sustained cytokine local expression and infiltration by inflammatory cells, decreased production of growth factors, reduced cellular proliferation and extracellular matrix synthesis, and increased production of proteolytic enzymes (37). Delayed growth factor production leads to impaired angiogenesis, endothelial progenitor cell dysfunction, and fewer platelets, further reducing granulation tissue formation, which in turn decreases growth factor secretion in areas of wound healing (38). These results suggest that the local administration of the growth factors at an early stage of healing may promote fracture healing in diabetes mellitus.

In the diabetes group, there was a delayed recovery of the biomechanical parameters in the first 5 weeks following tibial fracture. The delayed recuperation of the biomechanical parameters appears to be associated with delayed expression of the growth factors TGF-β1 and BMP-2.
